# Electrospinning PLLA/PCL Blend Fibre-Based Materials and Their Biomedical Application: A Mini Review

**DOI:** 10.3390/polym17202802

**Published:** 2025-10-20

**Authors:** Chen Meng

**Affiliations:** Department of Chemical Engineering and Biotechnology, University of Cambridge, Cambridge CB3 0AS, UK; cm2309@cam.ac.uk

**Keywords:** electrospinning, fibres, poly (L-lactic acid), polycaprolactone, biomedical

## Abstract

Fibres play a crucial role in diverse biomedical applications, ranging from tissue engineering to drug delivery. Electrospinning has emerged as a simple and versatile technique for producing ultrafine fibres at micro- to nanoscale dimensions. Synthetic biopolymers are effective cues to replace damaged tissue in the biomedical field, both in vitro and in vivo applications. Among them, poly (L-lactic acid) (PLLA) is a renewable, environmentally friendly biopolymer material. Polycaprolactone (PCL) is a synthetic polymer with good biocompatibility and biodegradation characteristics. However, both electrospun PLLA and PCL fibres have their limitations. To overcome these shortcomings, electrospinning PLLA/PCL blend fibres has been the subject of many studies. This review discusses the different parameters for the electrospinning of PLLA/PCL-based fibres for biomedical applications. Furthermore, we also discuss how electrospun PLLA/PCL-based scaffolds can be modified or combined with other biomaterials, such as natural polymers and bioceramics, and examine their in vitro and in vivo applications in various tissue repair strategies.

## 1. Introduction

Electrospinning is a fibre production method that utilizes electric force to draw charged threads from polymer solutions or melts, resulting in nanofibres with diameters from the hundreds of nanometres to a few micrometres [1]. This process, sharing characteristics of electrospraying and traditional fibre spinning, eliminates the need for coagulation chemistry or high temperatures, making it suitable for polymers with large and complex molecules [2]. The technique finds applications in diverse fields, such as biomedical, filtration, and energy storage, owing to its ability to produce nanofibres with high surface area and porosity [3,4,5,6]. Challenges such as achieving consistent fibre diameters and scaling up for industrial production persist, driving ongoing research and development in the optimization of parameters and exploration of new materials [7]. Despite these challenges, electrospinning stands as a versatile and promising method with broad implications across various industries.

**Figure 1:**
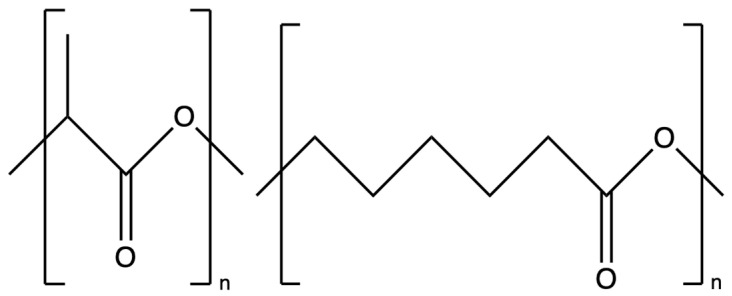
Chemical structures of PLLA (**left**) and PCL (**right**).

Bioresorbable polymer scaffolds have been developed to temporarily replace diseased tissues during the regeneration process, effectively eliminating the need for a second surgery. In recent years, synthetic polymers have garnered significant attention due to their unique advantages. The degradation products of these polymers are generally compatible with the metabolic pathways of the human body [8]. To date, over 100 types of natural and synthetic polymers have been electrospun into nano and microfibres. Table 1 shows some of the selected studies of electrospun natural/synthetic polymers. Among them, poly (L-lactic acid) (PLLA) is a synthetic biodegradable polymer that is widely used in the biomedical field, as shown in Figure 1 [9]. Moreover, PLLA has been approved by the Food and Drug Administration (FDA) for human usage in diverse applications, such as implantable medical devices, drug delivery carriers, and tissue regeneration scaffolds [10,11,12]. Polycaprolactone (PCL) is another one of the most commonly used synthetic polymers in biomedical applications, primarily due to its slow biodegradation rate and favourable biocompatibility [13,14,15]. Nonetheless, both PLLA and PCL have some limitations. Both PLLA and PCL exhibit certain limitations. For instance, PLLA has a glass transition temperature of approximately 60 °C, making it rigid under ambient and physiological conditions. Moreover, its inherent hydrophobicity hinders cell attachment, spreading, and proliferation. In addition, the higher density of ester groups along the PLLA polymer chain renders it more susceptible to hydrolysis, leading to a relatively rapid biodegradation rate. Compared with PLLA, PCL has some distinct differences in physical properties that attract researchers [16]. PCL is another FDA-approved biodegradable polyester, as shown in Figure 1. PCL is a semicrystalline polymer due to its regular molecular structure, with a melting temperature between 59 and 64 °C, which is above body temperature. Its glass transition temperature (Tg) is −60 °C, meaning that at body temperature, PCL remains in a rubbery state [17,18,19]. Moreover, owing to its semicrystalline structure and hydrophobic nature, PCL degrades at an exceptionally slow rate, typically requiring 2–4 years depending on its molecular weight [20]. This prolonged degradation profile makes PCL particularly attractive for long-term scaffold applications, such as tendon and ligament repair [21].

Recently, synthetic hybrid polymers proposed in biomedical materials have been the subject of many studies due to their versatile degradation rate, optimal porosity, and good resistance to high temperature and pressure, especially PLLA/PCL blend [31,32,33]. Blends of PLLA and PCL have been found to be immiscible, with PCL phases finely dispersed within the PLLA-rich phase [34]. Blending PLLA with PCL could reduce the tensile strength and Young’s modulus of PLLA. In PLLA/PCL blends, the elongation at break increases relative to PLLA alone, which is largely due to the plasticizing action of the amorphous mixed phase formed at the polymer interfaces [35]. More than that, the addition of PCL hindered the crystallization of PLLA, leading to a reduction in the crystallinity of the PLLA/PCL blends compared to pure PLLA. Therefore, the PLLA/PCL blends exhibited enhanced ductility [36]. Compared with pure PLLA, the PLLA/PCL blends resulted in thermal stability and active hydrolysis [33,37]. Furthermore, two separate degradation stages were observed in the PLLA/PCL blends, suggesting the presence of immiscible phases originating from the PLLA matrix and the dispersed PCL phase [35]. More than that, further evidence also highlights the profound influence of blending methodology on the final co-crystallization behaviour of PLLA/PCL systems. In the coalesced PLLA/PCL blend obtained via cyclodextrin-inclusion complexation, the PCL chains completely lost their crystallinity, while only a very small fraction (approximately 5%) of the PLLA chains remained crystalline [38]. Thus, adequately blending PLLA and PCL represents an innovative strategy for producing self-reinforced composites with customized properties tailored to specific biomedical applications.

Based on recent U.S. patent filings, the applications of electrospun nanofibres have been primarily focused on the field of biomedical prostheses, particularly in vascular grafts and tissue scaffolds. Notably, biodegradable polymer electrospun nanofibres have shown significant potential in life sciences and tissue engineering scaffolds [3]. Although several interesting reviews highlight the use of PLLA and PCL nanofibres for biomedical applications, there is a lack of reviews on the same topic for PLLA/PCL blend nanofibres [39,40,41,42]. This review highlights recent advances in electrospun PLLA/PCL nanofibres for biomedical applications. It outlines their current applications, summarizes key properties, and discusses modification strategies aimed at enhancing their performance. In addition, the review provides an overview of hybrid nanofibres that combine PLLA/PCL with other biomaterials, emphasizing fabrication approaches and property improvements, as well as prospects.

## 2. Electrospinning

**Figure 2:**
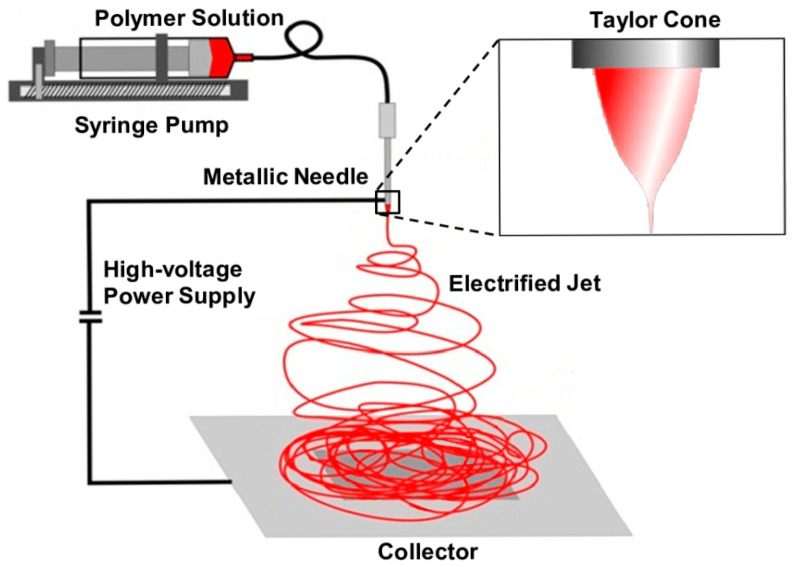
Schematic image of a traditional electrospinning unit, reproduced from Ref. [46].

Electrospinning can be traced back to 1887, with the technique gaining formal recognition in the 1960s through Geoffrey Taylor’s influential publications [43,44,45]. A standard electrospinning apparatus typically consists of a high-voltage DC or AC power supply, a syringe pump, a spinneret (usually a blunt hypodermic needle), and a collector, as shown in Figure 2. The electrospinning process begins with the ejection of a liquid droplet from the spinneret, which acquires an electric charge and elongates into a Taylor cone. Subsequently, a stream is expelled from the pinnacle of the Taylor cone, generating an elongated and slender strand of liquid. Initially, this strand travels straight, stretching and elongating within the electric field. Electrostatic repulsion within the strand eventually triggers bending instability at multiple points. As the strand extends, it coils into a sequence, resulting in a reduction in the fibre’s diameter. This decrease is primarily due to the coiling process. As the diameter diminishes, rapid solvent evaporation occurs, facilitating the solidification of the fibre, which subsequently accumulates on the collector.

The configuration parameters, such as voltage, distance from the collector, flow rate, and needle diameter, are highly customizable, allowing precise adjustment to achieve the desired fibre size. Fine-tuning the process parameters is crucial for ensuring stability in nanofibre production. These factors encompass the characteristics of polymer solutions (such as viscosity, conductivity, and volatility), environmental factors (including temperature and humidity), and electrospinning variables (such as flow rate, scan distance, electrical voltage, and collector rotation speed). The utility of polymer fibres is influenced by various factors, including diameter, surface features, and internal structures. A considerable amount of research has been dedicated to understanding critical factors that impact the structures of electrospun nanofibres. Firstly, in the preparation of the solution, extensive studies focus on the properties of the polymer solution. This involves exploring parameters like polymer concentration, viscosity, and the varying conductivity of different polymers [47]. Secondly, during the electrospinning process, specific setting parameters play a crucial role. These factors include the applied voltage, the distance from the capillary tip to the collector, the rotational speed of the collector (particularly if it is a metallic roller), and the injection rate of the syringe pump [48]. Each of these parameters significantly influences the outcome of the electrospinning process and requires careful optimization. Furthermore, the working environment also plays a vital role in shaping the morphologies of nanofibres. Factors such as temperature and humidity within the electrospinning unit need to be carefully controlled to achieve consistent and desired results [49]. In summary, the fabrication of target electrospun nanofibres necessitates meticulous attention to these main factors.

Electrospinning has been employed to produce nanofibres from a diverse range of materials. Among the most frequently utilized materials are organic/natural polymers, available either in solution or melt forms. Natural polymers are usually produced by living organisms. Being renewable resources, these materials offer excellent biocompatibility and biodegradability, rendering them environmentally friendly options. Numerous natural polymers have been successfully transformed into nanofibres, such as collagen, gelatin, silk fibroin and chitosan [50,51,52,53]. Compared to natural polymers, synthetic polymers are increasingly garnering attention from researchers due to their superior physical and chemical properties. Moreover, the ability of most synthetic polymers to dissolve in common solvents presents a significant advantage for electrospinning technology. As a result, a wide range of electrospun synthetic polymer fibres has been successfully developed and widely applied in various fields. The integration of sol–gel chemistry has enabled the direct electrospinning of various composite materials into nanofibres. The incorporation of nanoscale constituents with unique dimensions and shapes, including nanoparticles, nanorods, nanowires, nanotubes, and nanosheets, into polymer solutions has led to blends that are suitable for electrospinning.

Since its inception in the late 19th century and systematic development in the 1960s, electrospinning has become a key method for fabricating nanofibres. By precisely controlling solution, process, and environmental parameters, electrospinning enables the production of multifunctional nanofibres from natural or synthetic polymers, as well as composite materials incorporating nanostructures, demonstrating broad potential in biomedical and industrial applications.

## 3. Poly (L-Lactic Acid)

PLLA, an FDA-approved polymer, is recognized for its lower toxicity compared to other synthetic polymers [54]. Through in vivo and in vitro experiments, the anti-infective properties of PLLA have been confirmed, thereby promoting its application in the field of biomedical [55,56]. One significant advantage of PLLA over other biodegradable polymers lies in its performance during implantation, ensuring sufficient mechanical properties for extended regenerative processes [57]. Another crucial factor to consider when evaluating a material’s biological properties is the by-products produced during its biodegradation [57,58]. As PLLA undergoes degradation via hydrolysis, it produces lactic acid as a by-product, which is naturally occurring in the body and is eliminated as water and carbon dioxide through excretion [54]. PLLA is categorized as a resorbable synthetic polymer with a slow degradation rate, which is primarily due to the presence of an additional methyl group. This group enhances the hydrophobicity of the polymer and hence its resistance to hydrolysis degradation [59]. PLLA typically degrades in approximately 40 weeks in vitro and around 30 weeks in vivo [60,61].

As a biodegradable polymer approved by the FDA, PLLA is highly suitable for biomedical applications, especially tissue engineering scaffolds. There are various techniques for the fabrication of PLLA scaffolds, including additive manufacturing, electrospinning, phase separation, 3D printing, and particulate leaching [62,63,64,65]. Among them, electrospinning offers a convenient and inexpensive method for making pure PLLA scaffolds for tissue engineering. Moreover, electrospun PLLA scaffolds have a fibrous structure with diameters akin to those found in natural extracellular matrices [66,67]. Recently, these electrospun PLLA-based nanofibrous scaffolds have found wide use in biomedical applications such as wound dressings, drug delivery, tissue engineering scaffolds, and implants [68]. For instance, Ali Derakhshan et al. demonstrated that electrospun PLLA scaffolds hold great potential for bladder reconstruction or replacement. Their findings revealed that these scaffolds effectively support the growth and alignment of hBSMCs while exhibiting excellent cell compatibility, highlighting their promise in urinary bladder tissue engineering [69]. Moreover, Yang et al. reported that aligned PLLA nano/microfibrous scaffolds are suitable for neural tissue engineering. Their results demonstrated that neural stem cells elongate and extend neurites parallel to the orientation of the PLLA fibres. These findings suggest that aligned nanofibrous PLLA scaffolds could serve as promising cell carriers in neural tissue engineering [40]. In addition, Karanth et al. developed 3D-printed PLLA scaffolds for craniofacial bone regeneration. The findings revealed that these scaffolds had an elastic modulus comparable to trabecular bone, and the observed cell attachment and proliferation validated their excellent cytocompatibility [70]. Moreover, Das et al. fabricated an electrospinning piezoelectric PLLA nanofibrous scaffold for skin regeneration. The result shows that this piezoelectric PLLA scaffold under external ultrasound activation exhibits a good wound healing rate and a strong antibacterial effect [71].

PLLA is a biodegradable polymer with low toxicity, favourable mechanical properties, and excellent biocompatibility, making it highly suitable for biomedical applications. Through electrospinning, PLLA scaffolds with fibrous architectures resembling natural extracellular matrices can be fabricated, and such scaffolds have demonstrated promising applications and clinical potential across multiple fields.

## 4. Polycaprolactone

PCL stands out as one of the frequently employed synthetic polymers in biomedical applications. PCL, an aliphatic linear polyester, exhibits a Tg of approximately −60 °C and a Tm ranging between 59 and 64 °C. These thermal properties are influenced by its degree of crystallinity and molecular weight, the latter of which typically falls within the range of 3000 to 100,000 [20]. PCL is a biocompatible, bioresorbable, and cost-effective synthetic polymer. Owing to its semi-crystalline and hydrophobic characteristics, it degrades slowly, with a typical timespan of 2–4 years depending on the initial molecular weight. Its favourable mechanical properties further support its use across diverse biomedical applications [72,73]. Moreover, it has obtained FDA approval and has been utilized in clinical settings since the 1980s, primarily as a slow-release drug delivery device and suture material [16]. Compared with PLLA, PCL exhibits distinct differences in physical properties. Firstly, unlike PLLA, PCL does not possess isomers. PLLA exists in different forms, such as PDLA and combinations to form PDLLA, leading to distinct biological degradation and melting points. The melting point of PCL is notably lower than that of PLLA. This difference is likely a result of the higher polarity and potential for hydrogen bonding in PLLA when compared to PCL. In addition, PCL exhibits rheological and viscoelastic properties. Due to its low melting temperature, good blend compatibility, FDA approval, and cost-effectiveness, PCL holds broad potential for diverse applications [74].

Several studies have investigated the electrospinning of PCL. In biomedical applications, PCL fibres must be bead-free as fibre diameters should mimic the natural ECM morphology to facilitate optimal cell growth [13]. For instance, Li et al. employed disc-electrospinning to fabricate a three-dimensional scaffold composed of porous macro/nanoscale fibres. Their findings showed that the electrospun PCL scaffold not only promoted initial cell attachment but also markedly increased protein adsorption, supporting its potential as a 3D tissue engineering scaffold for soft tissue regeneration [75]. In addition, Safaeijavan et al. developed electrospun PCL nanofibres for cardiac tissue engineering. To improve the hydrophilicity of the scaffolds, plasma surface treatment was applied. Adipose-derived stem cells were subsequently cultured on scaffolds containing either random or aligned fibres to assess their differentiation into cardiomyocytes and overall biological performance. The results demonstrated that aligned scaffolds significantly promoted cardiomyogenic differentiation compared to random ones, indicating their strong potential for cardiac tissue reconstruction [76]. More than that, Meng et al. prepared a conjugate electrospun PCL micro-/nano-fibrous scaffolds. Results indicated that PCL micro-/nano-fibrous scaffolds were found to exhibit better mechanical properties and cell adhesion, and proliferation ratio than traditional PCL fibrous scaffolds [77].

Overall, PCL is a biocompatible, bioresorbable, and FDA-approved synthetic polymer, making it highly suitable for diverse biomedical applications. Electrospun PCL scaffolds have demonstrated enhanced cell adhesion, proliferation, and differentiation, highlighting their strong potential in tissue engineering fields such as soft tissue, cardiac, and regenerative medicine.

## 5. Electrospun PLLA/PCL

As mentioned above, PCL and PLLA are widely used in scaffold construction due to their excellent biocompatibility and non-toxic properties [78,79,80]. Additionally, PLLA is semi-crystalline, offering better mechanical strength and a controllable biodegradation capability [33,80,81]. PCL has more alkyl units between its ester bonds, giving it a more thermoplastic polymer chain, a lower crystallization temperature, and a longer biodegradation time [82]. Due to the high toughness of PCL, it was blended with PLLA to enhance the toughness of the PLLA scaffold without compromising its biodegradability [83]. Chen et al. investigated the mechanical and thermal properties of polymeric PLLA/PCL blend membranes with varying weight ratios (*w/w*%). The study revealed that increasing the PCL content led to a notable improvement in yield elongation (%). Moreover, incorporating PCL into PLLA enhanced the elongation at break (%). Appropriate amounts of PCL addition were also found to improve the elastic modulus (MPa), yield strength (MPa), and tensile strength (MPa) of the blends [84]. More than that, PLLA has better cell affinity than PCL [85]. The lack of functional moieties, such as carboxyl and -OR groups, on the PCL backbone, combined with its composition of an unsubstituted PE backbone terminated with carboxylic acids, slightly restricts its applicability as a surface-ready polymer for direct cell attachment [86]. The primary objective of tissue engineering is to design scaffolds that can replace damaged tissues, support cellular activity and controlled biodegradation during regeneration, and preserve mechanical integrity throughout the healing process [87]. On one hand, scaffolds need to stimulate physical and biochemical processes to promote tissue regeneration and provide the desired mechanical strength [88]. On the other hand, the degradation rate of the scaffold must match the rate of new tissue formation to maintain mechanical properties [89].

Using only one type of polymer to fabricate scaffolds often fails to meet all the characteristics required for specific clinical applications. Therefore, research has shifted toward studying blends that can enhance the fundamental properties of scaffolds [90]. Previous studies have demonstrated that combining PCL and PLLA within a single scaffold can integrate the mechanical flexibility of PCL with the excellent cell affinity of PLLA. The resulting PCL/PLLA scaffolds exhibited good cytocompatibility, with in vitro studies showing enhanced cell proliferation and metabolic activity [91,92]. For example, Can et al. conducted a study measuring the proliferation of human umbilical vein endothelial cells (HUVECs) in PLLA/PCL blends with varying weight ratios of 95/5, 90/10, 85/15, and 80/20. The results showed that on days 7 and 14, the cell population in all blends significantly increased compared to pure PLLA and PCL [93]. More than that, achieving strong, ductile mechanical properties alongside an optimized biodegradation rate can be accomplished by blending or copolymerizing PLLA with PCL.

Cardoso et al. did an initial study of electrospinning PCL/PLLA blends and investigated the effect of blend composition on fibre morphology, and successfully produced a fibrous mat with high porosity through electrospinning, demonstrating the potential application of electrospinning in fabricating scaffolds for tissue engineering [94]. After that, Lu et al. systematically investigated the effects of polymer blending ratios and mixed solvent compositions on fibre morphology [95]. The results show that the morphology of PLLA/PCL blend fibres is strongly dependent on the polymer blend ratio. Fibres with a lower PCL ratio exhibit better morphology than those with a higher PCL ratio, as the presence of PLLA enhances the solution viscosity. Additionally, using DMF as an auxiliary solvent further improves fibre morphology, as the strong polarity of DMF enhances the electrical conductivity of the electrospinning solution. In another study, Bolbasov et al. tested the morphological and biological properties of electrospun PCL, PLLA, and PCL/PLLA blend scaffolds. These scaffolds had similar average pore sizes of approximately 11.2 ± 5.0 μm. Moreover, the cell viability results showed that the electrospun PCL/PLLA blend scaffolds exhibited higher percentages of live cells on their surfaces compared to electrospun PCL scaffolds [82]. In addition, Madheswaran et al. reported a novel approach for continuously fabricating functional PLLA/PCL blend nanofibrous yarn through AC electrospinning and braiding technology. Results show that the final PLLA/PCL nanofibrous yarn exhibited excellent breaking force and thermal stability [96]. More than that, Oztemur et al. also showed that the addition of PLLA to PCL can enhance the cellular activity of the scaffold. Furthermore, they also investigated the degradation rate of PLLA/PCL, and the results showed that compared to pure PCL fibre webs, the biodegradation of the blend fibre webs with 50% PLLA increased from 3.7% to 7.69% [80]. In addition, Chen et al. explored the physical properties of electrospun nanofibrous scaffolds made from PLLA/PCL blends with different weight ratios, and their proliferation, attachment, viability, and multi-lineage differentiation were evaluated using human adipose-derived stem cells [97]. The results showed that all electrospun PLLA/PCL blend nanofibrous scaffolds could support hASCs effectively. The nanofibrous scaffold with a 1/1 weight ratio exhibited the best physical properties and cellular responses across all assessments, suggesting it to be a biocompatible scaffold for tissue engineering. Recently, our group developed a rapid and efficient method for fabricating highly porous PLLA/PCL fibrous scaffolds [98]. In this study, PLLA/PCL nanofibrous membranes were first fabricated via electrospinning and subsequently treated with acetone to create a highly porous structure. During this process, a portion of the PCL was partially extracted and enriched on the fibre surface. The porous PLLA/PCL scaffold exhibited a 250% increase in cell proliferation by day 10 compared with conventional PLLA/PCL scaffolds. These findings indicate that the porous PLLA/PCL nanofibrous membranes significantly promote osteoblast adhesion and proliferation.

Overall, blending PLLA with PCL effectively combines the mechanical strength and cell affinity of PLLA with the toughness and stability of PCL. Electrospun PLLA/PCL blend scaffolds show improved biodegradation, morphology, and cytocompatibility, highlighting their strong potential for tissue engineering applications, which represent a versatile and effective approach for designing next-generation biomimetic scaffolds with strong potential for clinical applications in tissue regeneration. Table 2 summarizes recent studies on electrospun PLLA/PCL blends.

## 6. Electrospun PLLA/PCL Hybrids

The PLLA/PCL blend system has garnered significant attention and is regarded as one of the most promising and extensively studied alternatives [97,106]. It exhibits numerous ideal properties for use in implants, including biocompatibility, bioresorbability, and biodegradability, while also showing encouraging results in clinical applications [107]. Although PLLA and PCL are biodegradable and biocompatible materials, electrospun PLLA/PCL fibrous membranes are still not good enough to be applied in tissue engineering due to weak bioactivity [108]. The reason is that both PLLA and PCL have low bioactivity, hydrophobicity, and prolonged degradation times in vivo [109]. To overcome the shortcomings, great efforts are being made to develop the composite fibres of PLLA/PCL and other natural/synthetic macromolecules via electrospinning for better properties. For example, Mashhadikhan et al. explored gelatin-coated PLLA/PCL hybrid scaffolds to enhance the mechanical properties and cytocompatibility [31]. The results showed that, compared with pristine PLLA/PCL scaffolds, gelatin-coated scaffolds exhibited a significant improvement in mechanical properties without altering the structural arrangement or fibre orientation that guides cell elongation. MTT analysis further revealed that gelatin coating markedly enhanced ADSC attachment, viability, and proliferation. In recent years, chitosan has also gained widespread recognition as a biomaterial owing to its excellent biocompatibility [110,111]. Especially, Chitosan could promote the growth of the dorsal root ganglion cell on the scaffold material [112]. Therefore, Matus-Munoz et al. fabricated scaffolds for epithelial cell differentiation based on electrospun chitosan, PCL, and PLLA blend composite fibres [113]. The results show that, after 24 h of culturing the immortalized human keratinocyte cell line HaCaT on the scaffold, the cells exhibited better proliferation and differentiation characteristics compared to the control group (PLLA/PCL fibrous scaffold).

Curcumin is a substance with anti-inflammatory, antimicrobial, antioxidant, and antifibrotic activities [114]. In the research reported by Doosti-Telgerd et al. curcumin-loaded PLLA/PCL fibre membranes were prepared using conjugated electrospinning technology. The results showed that, compared with PLLA/PCL fibrous membranes, curcumin-loaded fibrous membranes can effectively prevent postoperative adhesion in rat abdominal cavity and rabbit dura mater models by providing anti-inflammatory, antioxidant, and antibacterial properties as well as suppressing fibrosis through the downregulation of transforming growth factor-β1 expression. Furthermore, bone morphogenetic protein-2 (BMP-2) is well known for its ability to bind transmembrane receptors and activate bone-forming cells [115]. Wang et al. fabricated BMP-2 loaded bioactive PLLA/PCL nanofibrous scaffolds; the results showed that the PLLA/PCL nanofibrous scaffold loaded with BMP-2 exhibit superior cell proliferation capacity. Moreover, BMP-2 loaded PLLA/PCL nanofibrous scaffolds enhance bone tissue formation through the activation of the TGF-β/Smad2/3 signalling pathway [116].

Moreover, synthetic polymers such as poly(3-hydroxybutyrate-co-3-hydroxyvalerate) (PHBV), Poly lactic-co-glycolic acid (PLGA) and polyethylene glycol (PEG) have better biodegradability, biocompatibility and tissue responses when implanted in vivo [117,118]. For example, Kontogianni et al. fabricated a novel PLLA/PCL/PHBV polymer blend scaffold, and the cell viability assessment displays that the scaffold has excellent biocompatibility, allowing cells to proliferate [119]. More than that, Wang et al. fabricated a PLLA/PCL/PLGA porous bilayer nanofibre scaffold [120]. This porous bilayer nanofibrous scaffold mimics the microstructure of the ECM and features two distinct surfaces. The inner layer, with smaller pore sizes, prevents urine infiltration into the internal or transplanted cells while promoting the confluence of urethral epithelial cells along the lumen. In contrast, the outer layer, characterized by larger pores, provides sufficient space to support the infiltration and regeneration of vascular and smooth muscle cells [121]. Apart from that, in Sharifisamani et al.’s study, they prepared a PEG/PLLA/PCL composite yarn through electrospinning for the drug-loaded sheath, ensuring controlled drug release and enhanced suture performance [122].

In addition, the modification of electrospun aliphatic polyester fibres with inorganic nanomaterials has been explored for tissue engineering applications. For instance, antimicrobial PCL fibres were produced by electrospinning a PCL solution containing small amounts of silver-loaded zirconium phosphate nanoparticles, demonstrating potential for use in wound dressings [123]. More than that, halloysite nanotubes (HNTs) have also been added to the PLLA/PCL blend system [124]. Recent studies have reported that incorporating HNT into the PLLA polymer can mitigate the adverse effects of PLA’s acidic environment [125]. Given the widespread use of the PLLA/PCL system in implantable devices, incorporating halloysite nanotubes (HNTs) offers the potential to develop a novel drug delivery platform with sustained release capabilities, suitable for various applications in tissue engineering and drug delivery [124]. In addition, magnetic nanoparticles (MPs) are another type of inorganic material, which have low toxicity, superparamagnetic properties and good biocompatibility [126]. Haroosh et al. produced a novel electrospun hybrid composite material by mixing PLLA/PCL blends with MPs [127]. The results show that the addition of MPs to the PLLA/PCL blends significantly reduced the average fibre diameter and accelerated drug release. Among inorganic biomaterials, bioceramic materials are utilized in tissue engineering for their durability and compatibility with mineralized tissues like bone [128].

In the field of tissue engineering, Hydroxyapatite (HA), bioactive glasses (BG), and calcium phosphate (CaP) stand out as common bioceramic materials, serving as bioactive and biocompatible materials used as fillers for repairing bone defects [129]. Consequently, the integration of ceramic materials with polymers becomes valuable, as they can act as reinforcing agents and/or biomimetic signals, guiding cell differentiation and expediting the mineralization process [130]. HA is the primary inorganic component of the human bone [131]. It is frequently incorporated into scaffolds for bone regeneration due to its excellent biocompatibility, osteoconductive properties, and its function as a neutralizing agent stemming from its alkaline nature [132]. The inclusion of HA particles in scaffolds replicates the Ca–P-rich layer found in natural bone, thereby supplying essential ions for the mineralization process [133]. For example, Qi et al. prepared PLLA/PCL/HAP scaffolds that provided good properties to nanofibrous substrates [134]. The results demonstrated that PLLA/PCL/HAP scaffolds effectively promoted osteoblast proliferation, differentiation, and mineralization. HA-functionalized scaffolds exhibited superior osteoblast attachment and proliferation while further enhancing cellular differentiation. Amorphous calcium phosphate (ACP), typically serving as an intermediate phase in the formation of other calcium phosphate compounds, has been shown to possess excellent biocompatibility and bioresorbability, making it a highly promising material for artificial bone graft fabrication [135,136]. For example, in our group’s recent study, we focused on the incorporation of ACP nanoparticles into PLLA to produce electrospun PLLA/ACP fibrous membranes and generate a hierarchical porous structure [137]. The results show that the porous PLLA/ACP fibrous membrane demonstrated a significant improvement in cell adhesion and proliferation. BGs are inorganic materials based on the SiO_2_–CaO–P_2_O_5_ system, are being extensively investigated for bone tissue engineering. Under physiological conditions, they can induce the formation of a HA layer, which is the primary mineral component of bone, so that it could promote both osteoinduction and osteogenesis [138]. For example, Piatti et al. presented a work which incorporates the innovative sol–gel BGs in electrospun PCL fibres and evaluates this new glass nanoparticles with the polymeric system to understand the suitability of this composite material for hard and soft tissue engineering applications [139]. In addition, Canales et al. fabricated electrospun scaffolds based on PLLA, incorporating bioglass and zinc oxide nanoparticles to develop bifunctional biomaterials with enhanced bioactivity and antibacterial properties [138]. The results show the use of both n-BG and n-ZnO as fillers for the development of novel bifunctional PLLA scaffolds, offering combined bioactive and antibacterial properties for bone tissue engineering applications. However, previous studies have mostly focused on electrospinning blends of single polymers with BGs, and there has been no research to date on electrospun PLLA/PCL/BGs systems, which may represent a promising direction for future research.

PLLA/PCL blend system, while limited by low bioactivity and slow degradation when used alone, can be significantly enhanced through modification with natural polymers, bioactive molecules, inorganic nanoparticles, and bioceramics. Table 3 provides selective studies on electrospinning of PLLA/PCL blend-based hybrids. Such strategies improve mechanical strength, cytocompatibility, drug delivery, and osteogenic potential, making PLLA/PCL-based scaffolds highly promising for tissue engineering and regenerative medicine.

## 7. Electrospun PLLA/PCL-Based 3D Structure

Although electrospun PLLA/PCL nanofibrous scaffolds well suited for tissue engineering applications, the traditional electrospun fibrous scaffold has some limitations. Firstly, electrospinning PLLA/PCL nanofibrous scaffolds are usually 2D nanofibrous membranes [146,147]. These simply structured PLLA/PCL nanofibrous scaffolds fail to replicate the complex three-dimensional architecture of native tissues or organs [148]. Therefore, developing 3D PLLA/PCL nanofibrous scaffolds through electrospinning remains a challenging yet highly active area of research. Secondly, electrospun PLLA/PCL nanofibrous scaffolds stem from their topography and the morphological structure of the overlaid nanofibre mats, which typically exhibit apparent or equivalent pore sizes in the submicron range, electrospun PLLA/PCL nanofibrous scaffolds lack the essential macropores (ranging from tens to hundreds of micrometres) required for cell growth and tissue formation [149]. There are several traditional methods for 3D PLLA/PCL scaffolds preparation, including particle leaching, 3D printing, thermal induce phase separation and self-assembly, which are shown in Table 4. For example, Sadiasa et al. prepared 3D porous scaffolds composed of various ratios of PLLA and PCL through the salt leaching method for bone regeneration applications [150]. In vivo and in vitro results demonstrated enhanced bone formation with increased PLLA content in the scaffold, without inducing inflammation or immune responses. The scaffold shows potential for bone tissue engineering applications. In another study, Hassanajili et al. employed an indirect 3D printing approach to fabricate composite PLA/PCL/HA scaffolds, which exhibited significantly enhanced ALP activity and improved osteoblast function. These findings highlight the strong potential of the fabricated PLA/PCL/HA scaffolds for applications in bone tissue engineering [151]. Moreover, Qiu et al. prepared PLLA/PCL nanofibrous scaffold through the thermally induced phase separation (TIPS) method and dexamethasone (DEX) loaded aminated mesoporous silica (MSNs-NH2) nanoparticles were subsequently deposited on the PLLA/PCL scaffold by EPD. Nanoparticles were deposited by electrophoretic deposition [152]. Compared with BMSCs cultured on PLLA/PCL and MSNs-NH_2_/PLLA/PCL scaffolds, those grown on the DEX@MSNs-NH_2_/PLLA/PCL scaffold exhibited markedly enhanced osteogenic differentiation. Furthermore, in vivo experiments revealed that the DEX@MSNs-NH_2_/PLLA/PCL scaffold significantly accelerated calvarial defect healing compared with the conventional PLLA/PCL scaffold. However, these methods also have some drawbacks. Firstly, for the phase separation method, it is found that only a few materials can be fabricated into a nanofibrous scaffold by thermally induced phase separation [153], and that completely removing the solvents from the resulting scaffold always needs quite a long time [149]. For 3D printing techniques, using 3D printing technologies to fabricate scaffolds for tissue engineering does not have a similarity in morphology to that of native ECM, which is necessary for optimal cell attachment and proliferation [154]. Compared with these methods, electrospinning scaffolds could achieve many unique advantages. Firstly, electrospinning enables the fabrication of a porous fibrous network structure with exceptional interconnectivity between pores. This feature facilitates cell migration into the electrospun scaffold and the transport of nutrients [155]. Secondly, the electrospinning technique could generate fibres with diameters akin to those found in natural extracellular matrices, which can be employed across a broad spectrum of materials [67]. Furthermore, electrospun nanofibres offer the convenience of facile functionalization through the incorporation of bioactive substances like growth factors [149].

Therefore, it is important to develop electrospun 3D PLLA/PCL nanofibrous scaffolds with interconnected macropores and high porosities [156]. To achieve these requirements, combining micro- or nanometre-scale fibres produced via electrospinning with a natural sponge-like structure presents a highly promising strategy for developing nanofibrous scaffold systems for tissue engineering applications [157,158]. Yao et al. developed PLLA/PCL nanofibrous sponge scaffolds through a refined process [159]. This technique makes use of the low melting point of PCL and is specified as a thermally induced nanofibre self-agglomeration (TISA) [149]. Electrospun fabric membranes were broken down into shortened fibres using liquid nitrogen mortar. To ensure uniform fibre dispersion, repeated steps of grinding, sieving, and washing were performed. The nanofibres then self-assemble in heated water to form three-dimensional structures. Moreover, Mader et al. fabricated PLLA/PCL nanofibrous sponges by freeze-drying short nanofibre dispersions, followed by thermal annealing–induced physical crosslinking among the fibres [160]. The resulting 3D scaffolds exhibited excellent shape recovery, compressibility, and promoted both cell proliferation and infiltration. In our previous study, we developed a 3D fibrous scaffold composed of electrospun PLLA/PCL fibres [161]. This method allows precise control over the scaffold’s 3D geometry, even for complex structures. Notably, PCL within the fibres functions as an intrinsic binder, eliminating the need for additional binding or crosslinking agents. The resulting 3D fibrous scaffolds possess high porosity with well-organized, interconnected macropores. In vitro studies further demonstrated that scaffolds supported high cell viability and effective cell infiltration.

Traditional electrospun PLLA/PCL nanofibrous scaffolds are limited by their 2D morphology and lack of macropores. Recent advances such as TISA, freeze-drying, and fibre-based assembly have enabled the fabrication of 3D scaffolds with interconnected macropores, high porosity, and improved mechanical recovery, which are shown in Table 4 as well. These 3D PLLA/PCL nanofibrous scaffolds mimic native tissue architecture, supporting enhanced cell infiltration, proliferation, and differentiation, and thus represent a highly promising strategy for tissue engineering applications.

**Table 4:** Selective studies on 3D structure PLLA/PCL blend-based scaffolds.

Author	Year	Materials	Fabrication Method	Application	Reference
Hassanajili et al.	2019	PLLA, PCL and HA	3D printing	Bone tissue engineering, MG63 osteocarcinoma cells attachment, proliferation and ALP activity	[151]
Wang et al.	2021	PLLA and PCL	Electrospinning/hot pressing/supercritical CO2 batch foaming method.	Vascular patch, human umbilical endothelial cell adhesion and migration	[162]
Shahverdi et al.	2022	PLLA and PCL	Melt electrowriting	Mouse murine fibroblast and human umbilical vein endothelial cells attachment and proliferation	[163]
Guerra et al.	2018	PLLA and PCL	3D printing	Biodegradable stents, murine 3T3 fibroblast cells attachment and proliferation	[164]
Yao et al.	2017	PLLA and PCL	Electrospinning/thermally induced self-agglomeration	Bone tissue engineering, human mesenchymal stem cells, osteogenic differentiation, and bone formation in a mouse model	[159]
Sadiasa et al.	2013	PLLA and PCL	Salt leaching	Bone tissue engineering, MG63 osteoblast-like cells proliferation, bone formation in rabbit model	[150]
Samadian et al.	2020	PLLA, PCL, gelatin and taurine	Electrospinning/thermally induced phase separation	Bone tissue engineering, MG63 osteoblast-like cells proliferation, bone formation in mouse model	[165]
Meng et al.	2024	PLLA, PCL and bioactive glass	Electrospinning/progen leaching	Bone tissue engineering, human osteogenic sarcoma cells proliferation and infiltration	[161]
Qiu et al.	2016	PLLA, PCL, silica and dexamethasone	Thermally induced phase separation/electrophoretic deposition	Bone tissue engineering, primary rat bone marrow mesenchymal stem cells proliferation and differentiation, bone formation in mouse model	[152]
Dong et al.	2019	PLLA and PCL	Electrospinning with water bath collection	MG63 osteoblast-like cells proliferation and infiltration	[166]
Peiravi et al.	2025	PLLA, PCL and ZnO	3D printing	Osteoarthritis treatment, MG63 osteoblast-like cells proliferation and mineralization, cartilage tissue repair in rabbit model	[167]
Dhayer et al.	2025	PLLA and PCL	Melt-spinning/knitting	Adipose tissue reconstruction, murine bone marrow mesenchymal stem cells and preadipocytes cells differentiation, in vivo study with mouse model	[168]

## 8. Perspectives and Conclusions

Significant technological and scientific advancements have been achieved in the field of electrospinning for tissue repair and regeneration. In addition to its capacity to generate nanofibrous structures that closely mimic native extracellular matrices, electrospinning offers advantages such as ease of setup and flexibility in tuning the composition and morphology of the fibres. These features have made it possible to tailor electrospun nanofibrous scaffolds for specific tissue types. Among the materials used, aliphatic polyesters such as PLLA and PCL represent an important class of biodegradable and biocompatible polymers with broad applicability in both biomedical and conventional material fields.

PLLA/PCL blends can combine the high strength of PLLA with the excellent flexibility of PCL, allowing tunable mechanical properties, degradation rate, and processability. Due to these unique advantages, PLLA/PCL blend nanofibres have become a promising biomaterial for both soft and hard tissue repair. Many studies have focused on the fabrication of PLLA/PCL scaffolds, not only traditional electrospun fibrous membrane scaffolds but also novel three-dimensional nanofibre scaffolds combined with various post-processing techniques. Thus, electrospun PLLA/PCL nanofibres play a significant role in both in vitro and in vivo models of tissue engineering, and this review explores the latest advances of electrospun PLLA/PCL-based scaffolds in the biomedical field.

However, both PLLA and PCL do not possess any inherent biological activity and cannot play an active role in the healing process when applied as tissue scaffolds. Therefore, it is necessary to focus on optimizing processing parameters and developing hybrid materials that combine PLLA and PCL with other materials with biological activity, such as natural biomolecules, bioceramics or nanoparticles to enhance biological performance. However, to date, few studies have addressed how to further enhance the bioactivity of electrospun PLLA/PCL blend materials, which represents an important direction for future research. Furthermore, scaffolds that exhibit functional properties in vitro may cause adverse effects in vivo, as the in vivo environment encompasses all the biological and physical stimuli that dynamically change during the stages of tissue repair. Therefore, the clinical feasibility of electrspun PLLA/PCL-based scaffolds should be evaluated through more in vivo studies and clinical trials in the future.

## Figures and Tables

**Table 1:** Selective studies on electrospinning of natural and synthetic polymers.

Author	Year	Materials	Application	Reference
Yang et al.	2024	Silk fibroin/fibrin	Artificial blood vessels	[22]
Dieterle et al.	2022	Gelatin/Hydroxyapatite	Periodontal tissue engineering	[23]
Puigmal et al.	2023	Polyvinyl alcohol/Chitosan	Skin tissue engineering	[24]
Norouzi et al.	2022	Sodium alginate/Polycaprolactone	Novel nanofibrous biomaterial	[25]
Kenawy et al.	2022	Polyvinyl alcohol/Dextran	Wound healing	[26]
Zhang et al.	2024	Silk fibroin/Polyglycolic acid	Bone regeneration	[27]
Guzmán-Soria et al.	2023	Poly lactic-co-glycolic acid/Collagen	Tissue engineering	[28]
Virijević et al.	2024	Polycaprolactone/Polyethylene glycol	Wound Healing	[29]
Mustafa et al.	2024	Polycaprolactone/Polyethene oxide	Drug delivery	[30]

**Table 2:** Selective studies on electrospinning of PLLA/PCL blends.

Author	Year	Solvent System	Application	Reference
Behtaj et al.	2021	Chloroform/Ethanol (9/1)	Retinal progenitor cell proliferation	[86]
Oztemur et al.	2023	Chloroform/Ethanol/Acetic acid (8/1/1)	Analysis of morphological, chemical and thermal properties	[99]
Bolbasov et al.	2018	Hexafluoro-2-propanol	Fat-derived multipotent mesenchyme stem cells cell proliferation	[82]
Oztemur et al.	2023	Chloroform/Ethanol/Acetic acid (8/1/1)	Fibroblast cells and human umbilical endothelial cells proliferation	[80]
Vida et al.	2017	Chloroform and Chloroform/Acetone (4/1)	Vero cells and fibroblastic cells proliferation	[100]
Li et al.	2015	Chloroform	MC3T3-E1 cells proliferation	[101]
Meng et al.	2023	Dichloromethane/ Dimethylformamide (19/1)	Bone tissue scaffold, human osteogenic sarcoma cells proliferation	[98]
Lui et al.	2015	1,1,1,3,3,3-hexafluoro-2-propanol	Tendon regeneration, naproxen sodium release, L929 murine fibroblast cells proliferation	[102]
Shakhssalim et al.	2013	Chloroform/N, N-dimethylformamide (10/1)	Bladder reconstruction, human bladder smooth muscle cells proliferation	[103]
Liao et al.	2010	Chloroform/Methanol (3/1)	Adipose-derived stem cells proliferation and differentiation	[104]
Mobarra et al.	2018	Chloroform/N, N-dimethylformamide (8/2)	Diabetes mellitus therapy, human-induced pluripotent stem cells differentiation to beta islet-like cluster cells	[105]
Chen et al.	2013	Chloroform/Methanol (3/1)	Human adipose-derived stem cells proliferation and differentiation	[97]
Lu et al.	2012	Chloroform/ Dimethylformamide (4/1)	Analysis of surface morphology, phase structure, and hierarchical structures within the fibres	[95]

**Table 3:** Selective studies on electrospinning of PLLA/PCL blend-based hybrids.

Author	Year	Type of Addition	Addition Constituents	Application	Reference
Qi et al.	2016	Bioceramic	Hydroxyapatite particles	Bone tissue engineering, mouse calvaria-derived pre-osteoblastic cells proliferation, differentiation and mineralization of osteoblasts	[134]
Liao et al.	2012	Bioceramic	Hydroxyapatite particles	Higher porosity, higher hydrophilic properties and higher biodegradation properties	[140]
De Siqueira et al.	2019	Bioceramic	Hydroxyapatite particles	Osteoblast cells adhesion and proliferation	[141]
Mashhadikhan et al.	2015	Natural Molecule	Gelatin	Adipose Derived Stem Cells attachment, viability and proliferation	[31]
Jiang et al.	2017	Natural Molecule	Tannin	Skin tissue engineering, Neonatal human dermal fibroblast cells viability and proliferation	[108]
Matus-Munoz et al.	2022	Natural Molecule	Chitosan	Human keratinocyte cells proliferation and differentiation	[113]
Kalvand et al.	2023	Natural Molecule	Chitosan/Dextran/TGF-β1	Cartilage tissue engineering, mesenchymal stem cells differentiation	[142]
Wang et al.	2025	Natural Molecule	BMP-2	Bone tissue engineering, rat bone marrow-derived mesenchymal stem cells proliferation and osteogenic differentiation	[116]
Liao et al.	2024	Natural Molecule	Curcumin	Prevent postoperative adhesion, inhibit fibroblast adhesion, proliferation, and differentiation	[143]
Xu et al.	2019	Natural Molecule/Synthetic Material	Chitosan/Polypyrrole	Nerve repair and regeneration, PC12 cells differentiation, neurite growth and alignment	[111]
Liao et al.	2012	Synthetic Material	Multiwalled carbon nanotube	Adipose-derived stem cells proliferation and reorientation	[144]
Liao et al.	2012	Synthetic Material	Poly (ethylene glycol)	Adipose Derived Stem Cells attachment, viability and proliferation	[145]

## Data Availability

No new data were created or analyzed in this study. Data sharing is not applicable to this article.
